# Albumin-Based Inflammatory–Nutritional Indices as Novel Biomarkers for Severity Stratification and Re-Hospitalization Risk in Hyperemesis Gravidarum: A Retrospective Case–Control Study

**DOI:** 10.3390/biomedicines14010197

**Published:** 2026-01-16

**Authors:** Gülay Balkaş, Sümeyye Ünsal, Okan Oktar, Mustafa Can Akdogan, Murat Gözüküçük, Yusuf Üstün

**Affiliations:** 1 Department of Perinatology, Ankara Training and Research Hospital, 06230 Ankara, Turkey; 2 Department of Obstetrics and Gynecology, Ankara Training and Research Hospital, 06230 Ankara, Turkey; sumeyyeunsalgal@gmail.com (S.Ü.); drmustafacanakdogan@gmail.com (M.C.A.); dr.muratgozukucuk@gmail.com (M.G.); yusuf.ustun@sbu.edu.tr (Y.Ü.); 3 Department of Gynecologic Oncology, Ankara Training and Research Hospital, 06230 Ankara, Turkey; oknoktar@gmail.com

**Keywords:** hyperemesis gravidarum, albumin-based inflammatory–nutritional indices, m-HALP score, prognostic biomarkers, severity stratification, re-hospitalization

## Abstract

**Background**: The aim of this study was to evaluate the diagnostic and prognostic performance of albumin-based inflammatory–nutritional indices in hyperemesis gravidarum (HG) and to determine their associations with disease severity and risk of re-hospitalization. **Methods**: This retrospective case–control study included 246 women with HG and 246 gestational-age-matched healthy pregnant controls at 6–16 weeks of gestation. Disease severity was classified as mild, moderate, or severe using the Pregnancy-Unique Quantification of Emesis (24 h scale) (PUQE-24) score. A comprehensive panel of albumin-based inflammatory indices—including C-reactive protein-to-albumin ratio (CAR), fibrinogen-to-albumin ratio (FAR), neutrophil-to-albumin ratio (NAR), leukocyte-to-albumin ratio (LAR), neutrophil percentage-to-albumin ratio (NPAR), monocyte-to-albumin ratio (MAR), hemoglobin–albumin–lymphocyte–platelet (HALP) score, modified HALP (m-HALP) score, prognostic nutritional index (PNI) score, systemic immune-inflammation index-to-albumin (SII/Alb), and systemic inflammatory response index-to-albumin (SIRI/Alb)—was calculated from routine complete blood count and serum biochemistry results obtained at diagnosis. Receiver operating characteristic analysis, along with univariate and multivariate logistic regression models, was performed to evaluate diagnostic performance and identify predictors of severe HG and re-hospitalization. **Results**: Albumin-based indices exhibited severity-associated alterations, with an overall trend toward worsening immuno-nutritional status across increasing HG severity. Among these, m-HALP score demonstrated the strongest inverse correlations with PUQE-24 score, ketonuria grade, length of hospital stay, and re-hospitalization risk (r = −0.74 to −0.52; all *p* < 0.001) and achieved the highest discriminative accuracy for both severe HG (AUC 0.864, 95% CI 0.836–0.892, *p* < 0.001) and re-hospitalization (AUC 0.722, 95% CI 0.675–0.766, *p* < 0.001). In multivariable analysis, higher HALP, m-HALP, and PNI were independently associated with a lower likelihood of severe HG. For re-hospitalization, higher m-HALP and HALP were independently associated with a lower risk, whereas higher NPAR, higher ketonuria grade, and higher PUQE-24 score were independently associated with an increased risk of re-hospitalization. **Conclusions**: Albumin-based indices, particularly m-HALP, demonstrated robust diagnostic and prognostic performance in HG compared with conventional biomarkers. These readily available, cost-neutral composite biomarkers enable objective severity stratification and accurate identification of patients at elevated risk of recurrent hospitalization, offering immediate potential to guide personalized, evidence-based clinical management.

## 1. Introduction

Nausea and vomiting affect up to 90% of pregnancies and typically follow a mild, self-limited course [1]. In 0.3–10.8% of cases, however, symptoms progress to hyperemesis gravidarum (HG), a severe and debilitating disorder characterized by intractable vomiting, dehydration, electrolyte imbalance, significant weight loss (>5% of pre-pregnancy body weight), metabolic disturbances, and, in extreme cases, Wernicke encephalopathy, hepatic dysfunction, or esophageal rupture [2,3,4]. HG remains one of the leading indications for hospitalization in the first trimester and imposes considerable physical, psychological, and socioeconomic burden [5]. Established risk factors for HG include younger maternal age, nulliparity, low or high pre-pregnancy body mass index (BMI), multiple gestation, molar pregnancy, female fetal sex, history of HG in previous pregnancies, and conception via assisted reproductive technologies [6,7]. Emerging data also implicate psychosocial stressors, anxiety, and altered hypothalamic–pituitary–adrenal axis regulation in the exacerbation of symptoms [8,9].

Untreated or inadequately managed HG is associated with increased maternal morbidity—including anemia, venous thromboembolism, gestational hypertension, preeclampsia, and placental abruption—as well as adverse perinatal outcomes such as preterm birth and low birth weight [10,11]. Consequently, early identification of at-risk patients, accurate severity stratification, and elucidation of underlying pathophysiological mechanisms are critical to enable timely intervention and improve maternal–fetal prognosis [12].

The pathogenesis of HG remains incompletely understood. Proposed mechanisms include hormonal dysregulation (particularly involving human chorionic gonadotropin and estrogens), immune system activation, gastrointestinal dysmotility, *Helicobacter pylori* infection, altered carbohydrate and lipid metabolism, autonomic nervous system dysfunction, nutrient deficiencies, and genetic predisposition [13,14,15]. However, no single pathway fully explains the clinical heterogeneity of HG, supporting a multifactorial model in which hormonal, immunological, neurogastrointestinal, and metabolic disturbances interact synergistically [16,17].

Increasing evidence highlights systemic inflammation, oxidative stress, and nutritional depletion as central components of HG pathophysiology [18,19]. Numerous studies have reported elevated proinflammatory cytokines (e.g., IL-6, TNF-α), acute-phase reactants, and hematologic inflammatory indices in affected women, reinforcing the concept of inflammation-driven maternal physiological perturbation [20,21,22]. Concurrently, malnutrition—manifested by hypoalbuminemia, electrolyte imbalance, and micronutrient deficits—has been consistently associated with greater disease severity and prolonged hospitalization [22,23]. Malnutrition further exacerbates immune dysregulation, impairs hepatic protein synthesis, and perpetuates a vicious cycle of inflammation and catabolism [23,24,25].

In recent years, albumin-based inflammatory and nutritional indices have garnered considerable attention in obstetric research due to their ready availability, low cost, and robust prognostic performance across pregnancy-related disorders [26,27]. By integrating acute-phase reactants, hematologic parameters, and serum albumin—a key negative acute-phase protein and marker of nutritional reserve—these composite indices provide a holistic reflection of the interplay between systemic inflammation and malnutrition. The C-reactive protein-to-albumin ratio (CAR), fibrinogen-to-albumin ratio (FAR), neutrophil-to-albumin ratio (NAR), leukocyte-to-albumin ratio (LAR), monocyte-to-albumin ratio (MAR), and neutrophil percentage-to-albumin ratio (NPAR) collectively capture heightened inflammatory activity against the backdrop of declining albumin, thereby serving as sensitive indicators of inflammatory–nutritional imbalance [28,29,30,31,32]. Similarly, systemic immune-inflammation index-to-albumin (SII/Alb) and systemic inflammatory response index-to-albumin (SIRI/Alb) ratios incorporate multiple leukocyte subsets and platelet counts, offering a broader representation of innate and adaptive immune dysregulation in the context of protein-energy depletion. The hemoglobin–albumin–lymphocyte–platelet (HALP) score, its modified variant (m-HALP), and the prognostic nutritional index (PNI) further integrate anemia, lymphocyte-mediated immunity, and visceral protein status, yielding comprehensive metrics of immune-nutritional competence [33,34,35,36,37,38,39,40].

Although individual inflammatory biomarkers have been investigated in HG, most prior studies have focused on isolated parameters, thereby neglecting the concurrent nutritional compromise that profoundly influences clinical severity, treatment response, and maternal–fetal outcomes. Integrated albumin-based indices, by simultaneously reflecting inflammatory burden and nutritional impairment, may therefore provide enhanced diagnostic and prognostic accuracy compared with conventional markers.

Accordingly, the present study aimed to evaluate a comprehensive panel of these novel inflammatory–nutritional indices in patients with HG and gestational-age-matched controls. We sought to determine their diagnostic performance in distinguishing HG from physiologic pregnancy, their associations with established clinical severity measures Pregnancy-Unique Quantification of Emesis (24 h scale) (PUQE-24) score [41], ketonuria, hospitalization duration, and re-hospitalization), and their independent predictive value for severe disease and re-hospitalization, thereby contributing to a more nuanced understanding of HG pathophysiology and facilitating risk stratification in clinical practice.

## 2. Materials and Methods

### 2.1. Study Design and Eligibility Criteria

This retrospective case–control study was conducted in the Department of Perinatology at a tertiary referral center between January 2023 and November 2025. The study protocol adhered to the principles of the Declaration of Helsinki and received approval from the Institutional Ethics Committee (approval number: 25-708, dated 3 December 2025). Owing to the retrospective study design, the requirement for written informed consent was waived in accordance with institutional data-protection regulations.

Eligible participants were women aged 18–45 years with singleton pregnancies at 6–16 weeks of gestation. The study cohort comprised both outpatient and inpatient cases, categorized as patients with a confirmed diagnosis of HG or gestational-age-matched healthy pregnant controls without nausea/vomiting requiring medical intervention. Exclusion criteria included multiple gestation, molar pregnancy, pre-existing chronic hypertension, pregestational diabetes mellitus, chronic or active viral or autoimmune hepatitis, urinary tract infection, pre-existing gastrointestinal disorders, eating disorders (including anorexia nervosa and bulimia nervosa), pre-pregnancy body mass index (BMI) ≥ 35 kg/m^2^, known major fetal structural or chromosomal anomalies, and incomplete medical records.

### 2.2. Data Collection

All data were retrieved from the institutional electronic medical records and obstetric database. Pregnancy was confirmed by transvaginal ultrasonography demonstrating a viable intrauterine gestational sac. Gestational age was determined from the last menstrual period when reliable and confirmed or corrected by crown–rump length measurement at the first-trimester ultrasound examination. The following clinicodemographic and laboratory parameters were systematically collected for each participant: maternal age, gravidity, parity, pre-pregnancy BMI, gestational age at symptom onset and at diagnosis/enrollment, absolute weight loss during the index admission, duration of hospitalization, number of re-hospitalization episodes (irrespective of gestational age at re-admission), PUQE-24 score at presentation, spot urine ketonuria grade (0 to 4+ by dipstick), complete blood count parameters (hemoglobin, leukocyte, neutrophil, lymphocyte, monocyte, and platelet counts), thyroid-stimulating hormone (TSH) concentration, and serum concentrations of CRP, fibrinogen, and albumin.

### 2.3. Collection and Analysis of Blood and Urine Samples

All laboratory assays were conducted in accordance with standardized institutional protocols, either at the time of HG diagnosis or at the corresponding gestational age for controls. Venous blood samples were obtained at the initial outpatient visit for healthy controls and at the time of hospital admission for patients with HG, prior to the initiation of intravenous fluid replacement or antiemetic therapy. Concurrently, midstream urine samples were obtained for dipstick-based ketonuria assessment. All hematological and biochemical measurements were performed in the central laboratory of our tertiary referral center using standardized procedures and fully automated analyzers in accordance with institutional quality-control protocols.

The following albumin-based inflammatory and nutritional indices were calculated: LAR = leukocyte count (×10^9^/L)/albumin (g/L), NAR = neutrophil count (×10^9^/L)/albumin (g/L), MAR = monocyte count (×10^9^/L)/albumin (g/L), FAR = fibrinogen (g/L)/albumin (g/L), CAR = CRP (mg/L)/albumin (g/L), NPAR = [(neutrophil percentage)/albumin (g/L)] × 100, SII/Alb = [(neutrophil count × platelet count)/lymphocyte count]/albumin (g/L), SIRI/Alb = [(neutrophil count × monocyte count)/lymphocyte count]/albumin (g/L), HALP score = [hemoglobin (g/L) × albumin (g/L) × lymphocyte count (×10^9^/L)]/platelet count (×10^9^/L), m-HALP score = [hemoglobin (g/L) × albumin (g/L) × lymphocyte count (×10^9^/L) × platelet count (×10^9^/L)], PNI = 10 × albumin (g/dL) + 0.005 × lymphocyte count (/mm^3^). For indices requiring different units, laboratory values were converted as appropriate to ensure consistency with original formula definitions.

Urine samples from patients with HG were assessed for ketonuria using the dipstick method. Ketonuria severity was categorized into five grades based on dipstick readings: negative (0 mg/dL), 1+ (mild; 0.5–15 mg/dL), 2+ (moderate; 15–40 mg/dL), 3+ (marked; 40–80 mg/dL), and 4+ (severe; >80 mg/dL).

### 2.4. Diagnostic Criteria and Severity Assessment of Hyperemesis Gravidarum

HG was diagnosed in pregnant women presenting with persistent vomiting occurring more than three times per day, accompanied by weight loss exceeding 5% of pre-pregnancy body weight (or >3 kg) and ketonuria (≥1+ on dipstick testing), in the absence of any other identifiable medical, surgical, or gynecological cause [4,7].

Disease severity was evaluated using the PUQE-24 scoring system, which assesses symptoms over the preceding 24 h period across three domains: duration of nausea, number of vomiting episodes, and number of retching (dry heave) episodes (Table 1). The total score ranges from 3 to 15, with higher values indicating greater severity. Patients were categorized into three severity groups according to established cut-offs: mild (score 3–6), moderate (score 7–12), and severe (score 13–15) (Table 1). This classification was applied to all patients with HG at the time of initial evaluation and was used for subsequent subgroup analyses.

### 2.5. Statistical Analysis

Statistical analyses were performed using IBM SPSS Statistics for Windows, version 26.0 (IBM Corp., Armonk, NY, USA). Continuous variables were summarized as mean ± standard deviation (SD) for normally distributed data and as median with interquartile range (IQR) for non-normally distributed data. Normality of distributions was assessed using the Shapiro–Wilk test.

Between-group comparisons of continuous variables were conducted using the independent-samples *t*-test or one-way analysis of variance (ANOVA) with Tukey’s post hoc test for normally distributed data, and the Mann–Whitney U test or Kruskal–Wallis test followed by Bonferroni-adjusted pairwise comparisons for non-normally distributed data. Categorical variables were compared using the chi-square test with Bonferroni correction applied for multiple comparisons. Correlations between clinical severity parameters (PUQE-24 score, ketonuria grade, length of hospital stay, and re-hospitalization) and inflammatory–nutritional indices were evaluated using Spearman’s rank correlation coefficient. The diagnostic performance of the inflammatory–nutritional indices for discriminating severe HG and predicting re-hospitalization was evaluated using receiver operating characteristic (ROC) curve analysis. The area under the curve (AUC) with 95% confidence intervals (CI), optimal cut-off values, sensitivity, and specificity were reported.

To identify independent predictors of severe HG and re-hospitalization, univariate and multivariate binary logistic regression analyses were performed. Variables with *p* < 0.10 in univariate analysis or considered clinically relevant were included into the multivariate models. Adjusted covariates comprised maternal age, pre-pregnancy BMI, gravidity, parity, smoking status, gestational age at symptom onset, TSH level, and prior history of HG. Re-hospitalization frequency was descriptively reported; however, for inferential analyses, re-hospitalization was modeled as a binary outcome (any re-admission vs. none), as the retrospective nature of the data precluded reliable time-to-event or count-based modeling. Model calibration was assessed using the Hosmer–Lemeshow goodness-of-fit test, multicollinearity was evaluated using variance inflation factors (VIFs), and model explanatory power was quantified using the Nagelkerke R^2^ statistic. All tests were two-sided, and a *p* value < 0.05 was considered statistically significant.

## 3. Results

A total of 492 pregnant women were enrolled: 246 patients with HG and 246 gestational-age-matched healthy controls. Maternal demographic and clinical characteristics are presented in Table 2. Patients with HG had significantly lower BMI (*p* = 0.013), a higher prevalence of smoking (*p* = 0.001), and a markedly higher prevalence of prior HG (*p* = 0.001). Median TSH levels were significantly lower in the HG group compared with controls (0.68 vs. 1.58 mU/L; *p* = 0.001).

Based on PUQE-24 scores, the HG cohort was stratified into mild (*n* = 123), moderate (*n* = 71), and severe (*n* = 52) subgroups. Clinical severity markers and laboratory parameters across the four study groups are summarized in Table 3. Length of hospital stay, weight loss, PUQE-24 score, and ketonuria grade increased progressively with disease severity (all *p* < 0.001). The proportion of patients experiencing ≥3 re-hospitalization episodes was 12.1%, 16.9%, and 53.8% in the mild, moderate, and severe HG groups, respectively (*p* < 0.001). Among hematologic and biochemical parameters, platelet and neutrophil counts, fibrinogen levels, and CRP concentrations increased, whereas lymphocyte count and serum albumin levels decreased significantly with worsening HG severity.

Albumin-based inflammatory and composite hematologic indices are presented in Table 4. CAR, FAR, NAR, NPAR, MAR, SII/Alb, and SIRI/Alb were significantly elevated, whereas HALP, m-HALP, and PNI were significantly lower in association with increasing HG severity (all *p* ≤ 0.05).

Spearman correlation analysis (Table 5) revealed strong inverse correlations between HALP, m-HALP, PNI, and albumin and all clinical severity measures—including PUQE-24 score, ketonuria grade, length of hospital stay, and re-hospitalization (r = −0.74 to −0.40; all *p* < 0.001). In contrast, CAR, FAR, NAR, LAR, NPAR, MAR, SII/Alb, and SIRI/Alb showed significantly positive correlations with these parameters (r = +0.26 to +0.49; *p* ≤ 0.035).

The diagnostic performance of the inflammatory–nutritional indices for predicting severe HG was evaluated using ROC curve analysis (Table 6). Among the evaluated indices, m-HALP, HALP, and PNI demonstrated the highest predictive accuracy. For m-HALP, the optimal cut-off value of 2,227,179.07 yielded a sensitivity of 81.2% and a specificity of 78.4%, with an AUC of 0.864 (95% CI 0.836–0.892; *p* < 0.001). The optimal cut-off for HALP was 28.5, providing 78.5% sensitivity and 77.2% specificity, with an AUC of 0.845 (95% CI 0.815–0.875; *p* < 0.001). For PNI, an optimal cut-off of 44 yielded 72.1% sensitivity and 74.5% specificity, with an AUC of 0.811 (95% CI 0.776–0.846; *p* < 0.001).

The diagnostic performance of the inflammatory–nutritional indices for predicting re-hospitalization was assessed using ROC curve analysis (Table 7). Among the evaluated indices, m-HALP, HALP, and NPAR demonstrated the highest discriminative accuracy. For m-HALP, the optimal cut-off value of 2,312,179.13 yielded a sensitivity of 71.9% and a specificity of 66.8%, with an AUC of 0.722 (95% CI 0.675–0.766; *p* < 0.001). The optimal cut-off for HALP was 27.8, providing 69.9% sensitivity and 65.3% specificity, with an AUC of 0.701 (95% CI 0.652–0.748; *p* < 0.001). For NPAR, an optimal cut-off of 187 yielded 66.3% sensitivity and 72.1% specificity, with an AUC of 0.698 (95% CI 0.652–0.742; *p* < 0.001).

Univariate and multivariate logistic regression analyses (Table 8) demonstrated higher m-HALP (adjusted OR 0.47, 95% CI 0.31–0.70; *p* < 0.001), higher HALP (adjusted OR 0.52, 95% CI 0.34–0.79; *p* = 0.001), and higher PNI (adjusted OR 0.78, 95% CI 0.55–0.95; *p* = 0.012) were independently associated with a lower risk of severe HG after adjustment for maternal age, pre-pregnancy BMI, gravidity, parity, smoking status, gestational age at symptom onset, TSH level, and prior history of HG.

Univariate and multivariate logistic regression analyses (Table 9) demonstrated that higher m-HALP (adjusted OR 0.66, 95% CI 0.51–0.84; *p* = 0.001) and higher HALP (adjusted OR 0.71, 95% CI 0.56–0.90; *p* = 0.005) were independently associated with a lower risk of re-hospitalization. In contrast, higher NPAR (adjusted OR 1.33, 95% CI 1.01–1.77; *p* = 0.043), higher ketonuria level (adjusted OR 1.41, 95% CI 1.10–1.81; *p* = 0.006), and higher PUQE-24 score (adjusted OR 1.18, 95% CI 1.01–1.39; *p* = 0.041) were independently associated with an increased likelihood of re-hospitalization.

## 4. Discussion

HG is one of the most severe health problems of early pregnancy, with profound physical, psychological, and socioeconomic consequences. Although the pathogenesis of nausea and vomiting of pregnancy remains incompletely understood and is widely recognized as multifactorial, accumulating evidence suggests that HG is characterized by a systemic maternal inflammatory state rather than by isolated gastrointestinal or hormonal dysfunction [21,42]. This paradigm shift is supported by consistent observations of elevated circulating inflammatory mediators, including CRP, interleukin-6, tumor necrosis factor-α, and other cytokines, alongside neutrophil activation, acute-phase reactant upregulation, and placental transcriptomic signatures resembling innate immune activation [43,44].

Recent landmark genomic studies have identified growth differentiation factor-15 (GDF15) and insulin-like growth factor binding protein-7 (IGFBP7) as key genetic risk factors for HG. Placental overexpression of GDF15 activates its cognate brainstem receptor, the glial cell line-derived neurotrophic factor family receptor alpha-like (GFRAL), thereby inducing anorexia and emesis. This GDF15–GFRAL signaling pathway has also been implicated in the amplification of systemic inflammation and oxidative stress, providing a mechanistic link between placental signaling, central emetic pathways, and maternal inflammatory responses in HG. IGFBP7, which is involved in cellular stress responses and inflammatory regulation, may further contribute to this proinflammatory milieu, reinforcing the concept of HG as a disorder characterized by integrated placental, metabolic, and immune dysregulation [45,46].

Concomitantly, hypoalbuminemia emerges as one of the most consistent and clinically meaningful laboratory abnormalities observed in HG, with serum albumin concentrations declining in parallel with disease severity [24]. This reduction cannot be explained solely by hemodilution or urinary loss. Instead, it likely reflects a convergence of cytokine-mediated suppression of hepatic albumin synthesis, increased capillary permeability with extravasation into the interstitial compartment, and sustained catabolic consumption during prolonged negative energy balance [23,24,25,47]. Importantly, hypoalbuminemia may further perpetuate systemic inflammation by reducing oncotic pressure, exacerbating tissue edema, and promoting ongoing cytokine release, thereby establishing a self-reinforcing inflammatory–nutritional cycle. The coexistence of heightened systemic inflammation and profound nutritional depletion creates a distinctive pathophysiological milieu in HG, wherein traditional inflammatory markers rise while immuno-nutritional reserves decline [48,49,50,51]. This dual perturbation provides a strong biological rationale for the use of albumin-based inflammatory–nutritional indices that simultaneously capture both dimensions of disease burden.

Building on emerging evidence that integrated immuno-nutritional markers outperform isolated laboratory parameters in reflecting disease severity across inflammatory conditions, the present study evaluated a comprehensive panel of albumin-based indices in a large cohort of women with HG compared with gestational-age-matched healthy controls. Across these indices, severity-associated alterations were observed, with more pronounced inflammatory ratios and lower immuno-nutritional composite scores in patients with more severe clinical phenotypes. These changes demonstrated consistent associations with validated clinical measures, including PUQE-24 score, ketonuria grade, length of hospital stay, and re-hospitalization risk, supporting their relevance to clinically meaningful outcomes.

At the cellular level, increasing HG severity was characterized by lower serum albumin concentrations, reduced lymphocyte counts, and declining hemoglobin levels, accompanied by relative neutrophilia and thrombocytosis. This pattern was reflected in higher values of proinflammatory ratios (CAR, NAR, LAR, NPAR, SII/Alb, and SIRI/Alb) and generally lower values of composite immuno-nutritional indices (HALP, m-HALP, and PNI) in more severe disease. Among the evaluated indices, m-HALP demonstrated particularly strong associations with disease severity and adverse clinical outcomes. It showed robust inverse correlations with PUQE-24 score, ketonuria grade, length of hospitalization, and re-hospitalization, and achieved the highest discriminative accuracy for both severe HG and re-hospitalization risk.

In multivariable models adjusting for established clinical confounders, higher m-HALP, HALP, and PNI values were independently associated with a lower likelihood of severe HG, whereas proinflammatory ratios did not retain independent associations. For re-hospitalization, higher m-HALP and HALP values remained independently protective, while higher NPAR, ketonuria grade, and PUQE-24 score were independently associated with an increased risk.

To the best of our knowledge, this study is the first to evaluate a comprehensive panel of albumin-based inflammatory–nutritional indices across severity grades of HG and to characterize the performance of m-HALP score within this context. To date, no previous study has specifically examined the association between m-HALP and either disease severity or re-hospitalization risk in HG, thereby limiting direct comparison with existing literature.

The observed performance of m-HALP may be attributed to its capacity to integrate multiple complementary pathophysiological domains central to HG, including catabolic depletion reflected by hypoalbuminemia, immune dysregulation manifested as lymphopenia, erythropoietic suppression leading to anemia, and relative thrombocytosis potentially reflecting reactive inflammatory responses. By capturing this multidimensional inflammatory–nutritional axis within a single composite metric, m-HALP may provide a more comprehensive representation of overall disease burden than isolated acute-phase reactants or inflammation-dominant indices. This integrated profile may partly explain why individual inflammatory markers and inflammation-dominant composite indices did not retain independent predictive value in fully adjusted models, despite demonstrating significant associations in univariate analyses.

From a clinical perspective, m-HALP may offer immediate translational utility as a cost-neutral adjunct to symptom-based assessment tools such as PUQE-24. By integrating routinely available complete blood count parameters with serum albumin levels, m-HALP enables objective assessment of inflammatory and nutritional status and may help identify patients at increased risk of severe disease or recurrent hospitalization. This complementary, laboratory-based risk stratification may inform timely escalation from outpatient to inpatient management, guide decisions regarding the initiation of enteral or parenteral nutritional support, and support risk-adapted follow-up strategies. Collectively, such an approach has the potential to optimize resource utilization and reduce the substantial healthcare burden associated with HG, particularly in resource-limited settings where access to advanced diagnostic tools is constrained.

This study has several notable strengths, including its relatively large sample size, strict patient selection criteria, rigorous adjustment for clinically relevant confounders, and the novel evaluation of a comprehensive panel of albumin-based inflammatory–nutritional indices in HG. Nevertheless, several limitations warrant consideration. The retrospective, single-center design introduces potential selection bias and may limit the generalizability of the findings across different populations and healthcare settings. Reliance on routinely collected medical records precluded assessment of dynamic, longitudinal changes in inflammatory–nutritional indices and limited evaluation of treatment-related trajectories over time. Although extensive multivariable adjustment was performed, residual confounding from unmeasured factors—such as dietary intake, psychosocial stress, or symptom duration prior to presentation—cannot be fully excluded. In addition, ketonuria was assessed using dipstick testing rather than quantitative assays, which may have introduced measurement variability.

Despite these limitations, the present findings indicate that albumin-based immuno-nutritional indices, particularly m-HALP score, capture clinically meaningful dimensions of disease burden in HG. Incorporation of albumin-based immuno-nutritional indices into clinical practice may facilitate a shift in HG management from predominantly empiric supportive care toward a more personalized, evidence-based approach. Importantly, prospective, multi-center validation studies are now required to confirm the generalizability of these findings, establish standardized diagnostic thresholds, and determine whether m-HALP-guided management strategies can improve maternal outcomes and reduce healthcare utilization in this debilitating condition.

## 5. Conclusions

Albumin-based inflammatory–nutritional indices, particularly the m-HALP score, provide accurate, accessible, and cost-neutral measures of disease burden in HG. Among the evaluated indices, m-HALP demonstrated the strongest diagnostic and prognostic performance, reliably identifying patients at increased risk of severe disease and re-hospitalization using only routinely available laboratory parameters. These findings underscore the potential clinical value of incorporating m-HALP into standardized assessment pathways. Prospective validation in independent cohorts is now required to confirm its clinical utility and to determine whether m-HALP-guided management strategies can improve maternal outcomes in this debilitating condition.

## Figures and Tables

**Table 1:** Pregnancy-Unique Quantification of Emesis (24 h scale) scoring index.

On average, over a 24 h period, for how long do you feel nauseated or sick to your stomach?	Not at all (1)	≤1 h (2)	2 to 3 h (3)	4 to 6 h (4)	>6 h (5)
On average, over a 24 h period, how many times do you vomit or throw up?	I did not throw up (1)	1 to 2 times (2)	3 to 4 times (3)	5 to 6 times (4)	≥7 times (5)
On average, over a 24 h period, how many times do you experience retching or dry heaves without emesis?	None (1)	1 to 2 times (2)	3 to 4 times (3)	5 to 6 times (4)	≥7 times (5)

Mild = ≤6, Moderate = 7–12, Severe = 13–15.

**Table 2:** Maternal demographic and perinatal outcomes of the study population.

	Control Group (*n* = 246)	HG Group (*n* = 246)	*p*-Value
Maternal age (years) (mean ± SD)	27.8 ± 5.1	25.8 ± 3.2	0.546 ^a^
Gravida (mean ± SD)	2.12 ± 0.45	2.06 ± 0.53	0.718 ^a^
Parity (mean ± SD)	0.93 ± 0.36	0.75 ± 0.58	0.645 ^a^
BMI (kg/m^2^) (mean ± SD)	26.37 ± 5.67	24.43 ± 6.18	0.013 ^b^
Gestational age at onset (weeks) (mean ± SD)	9.56 ± 2.24	10.06 ± 1.42	0.255 ^a^
History of abortion (*n*, %)	15 (6)	17 (7)	0.385 ^c^
Smoking status (*n*, %)	28 (11.4)	43 (17.5)	0.001 ^c^
History of HG (*n*, %)	11 (4.48)	53 (21.54)	0.001 ^c^
TSH (mU/L) median (IQR)	1.58 (0.95–2.45)	0.68 (0.38–1.62)	0.001 ^b^

Abbreviations: BMI: body mass index; HG: hyperemesis gravidarum; TSH: thyroid-stimulating hormone; SD: standard deviation; ^a^ independent-samples *t*-test; ^b^ Mann–Whitney U; ^c^ chi-square test. *p* < 0.05 indicates statistical significance compared with the control group.

**Table 3:** Comparison of clinical characteristics, ketonuria distribution, and laboratory parameters across study groups.

	Control (*n* = 246)	Mild HG (*n* = 123)	Moderate HG (*n* = 71)	Severe HG (*n* = 52)	*p*-Value
Weight loss (kg)	0	0.09 ± 0.05	0.16 ± 0.07	0.35 ± 0.18	<0.001
Length of hospital stay (days)	0	2.75 ± 0.85	3.14 ± 1.25	5.25 ± 2.67	<0.001
Re-hospitalization	246 (100)				<0.001
0		28 (22.8)	8 (11.3)	0 (0)	
1		45 (36.6)	23 (32.4)	8 (15.4)	
2		35 (28.5)	28 (39.4)	16 (30.8)	
≥3		15 (12.1)	12 (16.9)	28 (53.8)	
PUQE-24 score	*n*/A	3.86 ± 0.54	8.57 ± 1.37	14.75 ± 1.25	<0.001
Ketonuria Level (Dipstick)					<0.001
0	246 (100%)	48 (39.0%)	11 (15.5%)	0 (0%)	
1+	0 (0%)	52 (42.3%)	24 (33.8%)	4 (7.7%)	
2+	0 (0%)	18 (14.6%)	21 (29.6%)	12 (23.1%)	
3+	0 (0%)	5 (4.1%)	11 (15.5%)	16 (30.8%)	
4+	0 (0%)	0 (0%)	4 (5.6%)	20 (38.4%)	
Hemoglobin (g/dL)	12.7 ± 0.9	12.3 ± 1.2	12.1 ± 1.1	11.9 ± 1.2	0.062
Platelet count (×10^9^/L)	249 (210–286)	276 (238–315)	292 (255–338)	318 (281–362)	0.042
Neutrophil count (×10^9^/L)	6.19 (5.25–7.13)	6.32 (5.14–7.50)	7.81 (6.42–9.20)	9.25 (7.63–10.87)	0.024
Lymphocyte count (×10^9^/L)	1.91 (1.74–2.08)	1.71 (1.31–2.11)	1.68 (1.35–2.01)	1.61 (1.32–1.90)	0.001
Albumin (g/L)	41 (38–44)	39 (34–42)	37 (32–38)	33 (28–34)	0.002
Fibrinogen (g/L)	3.82 (3.01–4.63)	3.23 (2.02–4.04)	3.81 (2.65–4.97)	4.44 (3.17–5.71)	0.001
CRP (mg/L)	3.2 (1.8–4.6)	6.9 (4.3–9.5)	12.5 (8.9–16.1)	18.9 (13.7–24.1)	<0.001

Abbreviations: HG: hyperemesis gravidarum; PUQE-24: Pregnancy-Unique Quantification of Emesis (24 h scale); CRP: C-reactive protein. Data are presented as mean ± SD, median (IQR), or *n* (%).

**Table 4:** Comparison of albumin-based inflammatory and composite hematologic indices across study groups.

	Control (*n* = 246)	Mild HG (*n* = 123)	Moderate HG (*n* = 71)	Severe HG (*n* = 52)	*p*-Value
CAR	0.078 (0.045–0.115)	0.181 (0.140–0.220)	0.357 (0.305–0.410)	0.609 (0.553–0.665)	0.012
FAR	0.093 (0.068–0.118)	0.085 (0.063–0.108)	0.109 (0.077–0.140)	0.143 (0.117–0.169)	0.001
NAR	0.151 (0.126–0.176)	0.166 (0.140–0.192)	0.223 (0.187–0.259)	0.298 (0.256–0.340)	<0.001
LAR	0.102 (0.090–0.114)	0.075 (0.065–0.085)	0.061 (0.052–0.070)	0.047 (0.040–0.054)	0.024
NPAR	157 (140–175)	184 (160–208)	223 (195–251)	298 (270–325)	<0.001
MAR	0.042 (0.035–0.048)	0.048 (0.040–0.056)	0.057 (0.049–0.065)	0.066 (0.060–0.072)	0.043
HALP score	33.4 (30.1–36.7)	28.9 (25.2–32.6)	26.3 (23.0–29.6)	21.2 (18.0–24.4)	<0.001
m-HALP score	2,476,397.56 (2,104,938.61–2,847,857.32)	2,227,179.32 (1,893,102.51–2,561,256.43)	2,286,991.18 (1,943,942.65–2,630,039.27)	2,179,499.46 (1,852,574.09–2,506,424.29)	<0.001
PNI score	49.1 (45.3–52.9)	44.5 (40.0–48.9)	41.0 (36.9–45.1)	36.6 (32.8–40.4)	<0.001
SII/Alb	19.7 (16.3–23.1)	26.8 (22.3–31.3)	38.8 (33.2–44.4)	59.0 (52.1–65.9)	<0.001
SIRI/Alb	0.136 (0.110–0.162)	0.177 (0.150–0.204)	0.265 (0.228–0.302)	0.379 (0.330–0.428)	<0.001

Abbreviations: HG: Hyperemesis Gravidarum; CAR: C-reactive protein-to-albumin ratio; FAR: Fibrinogen-to-albumin ratio; LAR: leukocyte-to-albumin ratio; NAR: neutrophil-to-albumin ratio; MAR: monocyte-to-albumin ratio; SII/Alb: systemic immune-inflammation index-to-albumin ratio; SIRI/Alb: systemic inflammatory response index-to-albumin ratio; NPAR: neutrophil percentage-to-albumin ratio; PNI: prognostic nutritional index; HALP: hemoglobin–albumin–lymphocyte–platelet; m-HALP: modified HALP. Values are presented as medians (IQRs).

**Table 5:** Correlation between clinical severity measures and inflammatory–nutritional indices in hyperemesis gravidarum.

	PUQE-24 Score	Ketonuria	Length of Hospital Stay	Re-Hospitalization	*p*-Value
HALP score	−0.65	−0.48	−0.72	−0.58	<0.001
m-HALP score	−0.67	−0.52	−0.74	−0.63	<0.001
PNI score	−0.59	−0.47	−0.69	−0.55	<0.001
Albumin	−0.44	−0.32	−0.40	−0.36	<0.001
CAR	+0.41	+0.36	+0.46	+0.40	0.002
FAR	+0.39	+0.34	+0.43	+0.38	0.013
NAR	+0.33	+0.28	+0.40	+0.31	<0.001
LAR	+0.31	+0.27	+0.35	+0.30	0.004
NPAR	+0.37	+0.31	+0.44	+0.36	<0.001
MAR	+0.29	+0.26	+0.33	+0.30	0.035
SII/Alb	+0.46	+0.37	+0.49	+0.42	<0.001
SIRI/Alb	+0.44	+0.35	+0.47	+0.41	0.003

Abbreviations: PUQE-24: Pregnancy-Unique Quantification of Emesis (24 h scale); CAR: C-reactive protein-to-albumin ratio; FAR: fibrinogen-to-albumin ratio; NAR: neutrophil-to-albumin ratio; LAR: leukocyte-to-albumin ratio; NPAR: neutrophil percentage-to-albumin ratio; MAR: monocyte-to-albumin ratio; HALP: hemoglobin–albumin–lymphocyte–platelet; m-HALP: modified HALP; PNI: prognostic nutritional index; SII/Alb: systemic immune-inflammation index-to-albumin ratio; SIRI/Alb: systemic inflammatory response index-to-albumin ratio. Values represent Spearman correlation coefficients (r). All correlations are statistically significant at *p* < 0.05.

**Table 6:** Receiver operating characteristic (ROC) analysis of inflammatory–nutritional markers for predicting hyperemesis gravidarum severity.

	Cut-Off Value	AUC	95% CI	Sensitivity	Specificity	*p*-Value
CAR	0.185	0.801	0.765–0.837	0.741	0.713	0.013
FAR	0.095	0.662	0.615–0.709	0.587	0.638	0.001
NAR	0.165	0.773	0.734–0.812	0.681	0.705	<0.001
LAR	0.065	0.572	0.520–0.624	0.554	0.521	0.024
NPAR	191	0.792	0.756–0.828	0.723	0.725	<0.001
MAR	0.050	0.641	0.594–0.688	0.543	0.624	0.002
HALP score	28.5	0.845	0.815–0.875	0.785	0.772	<0.001
m-HALP score	2,227,179.07	0.864	0.836–0.892	0.812	0.784	<0.001
PNI score	44.0	0.811	0.776–0.846	0.721	0.745	<0.001
SII/Alb	26.0	0.703	0.660–0.746	61	0.693	<0.001
SIRI/Alb	0.175	0.742	0.701–0.783	65	0.705	<0.001

Abbreviations: CAR: C-reactive protein-to-albumin ratio; FAR: fibrinogen-to-albumin ratio; NAR: neutrophil-to-albumin ratio; LAR: leukocyte-to-albumin ratio; NPAR: neutrophil percentage-to-albumin ratio; MAR: monocyte-to-albumin ratio; HALP: hemoglobin–albumin–lymphocyte–platelet; m-HALP: modified HALP; PNI: prognostic nutritional index; SII/Alb: systemic immune-inflammation index-to-albumin ratio; SIRI/Alb: systemic inflammatory response index-to-albumin ratio; AUC: area under the curve; CI: confidence interval.

**Table 7:** Receiver operating characteristic (ROC) analysis for predicting re-hospitalization in hyperemesis gravidarum.

	Cut-Off Value	AUC	95% CI	Sensitivity	Specificity	*p*-Value
CAR	0.22	0.612	0.558–0.662	63	0.593	0.003
FAR	0.11	0.522	0.468–0.576	41	0.570	0.412
NAR	0.18	0.64	0.589–0.690	0.663	0.604	<0.001
LAR	0.065	0.572	0.520–0.624	0.552	0.536	0.021
NPAR	187	0.698	0.652–0.742	0.663	0.721	<0.001
MAR	0.05	0.605	0.551–0.656	0.615	0.575	0.006
HALP score	27.8	0.701	0.652–0.748	0.699	0.653	<0.001
m-HALP score	2,312,179.13	0.722	0.675–0.766	0.719	0.668	<0.001
PNI score	42.5	0.683	0.632–0.731	0.626	0.709	<0.001
SII/Alb	30.5	0.588	0.535–0.641	0.602	0.557	0012
SIRI/Alb	0.19	0.621	0.568–0.671	0.632	0.583	0.001

Abbreviations: CAR: C-reactive protein-to-albumin ratio; FAR: fibrinogen-to-albumin ratio; NAR: neutrophil-to-albumin ratio; LAR: leukocyte-to-albumin ratio; NPAR: neutrophil percentage-to-albumin ratio; MAR: monocyte-to-albumin ratio; HALP: hemoglobin–albumin–lymphocyte–platelet; m-HALP: modified HALP; PNI: prognostic nutritional index; SII/Alb: systemic immune-inflammation index-to-albumin ratio; SIRI/Alb: systemic inflammatory response index-to-albumin ratio; AUC: area under the curve; CI: confidence interval.

**Table 8:** Logistic regression analyses of inflammatory–nutritional and hematologic markers for predicting severe hyperemesis gravidarum.

	Univariate OR (95% CI)	*p*-Value	Adjusted OR (95% CI) *	*p*-Value
CAR	2.84 (1.92–4.19)	<0.001	1.42 (0.91–2.21)	0.112
FAR	1.58 (1.10–2.28)	0.014	1.12 (0.73–1.72)	0.598
NAR	2.31 (1.64–3.25)	<0.001	1.38 (0.92–2.09)	0.118
LAR	0.85 (0.62–1.17)	0.321	—	—
NPAR	2.56 (1.81–3.63)	<0.001	1.51 (0.97–2.33)	0.068
MAR	1.44 (1.05–1.99)	0.026	1.09 (0.75–1.60)	0.644
HALP	0.41 (0.28–0.59)	<0.001	0.52 (0.34–0.79)	0.001
m-HALP	0.38 (0.26–0.55)	<0.001	0.47 (0.31–0.70)	<0.001
PNI	0.56 (0.42–0.76)	<0.001	0.78 (0.55–0.95)	0.012
SII/Albumin	1.88 (1.34–2.62)	<0.001	1.21 (0.85–1.72)	0.286
SIRI/Albumin	2.09 (1.48–2.96)	<0.001	1.33 (0.89–1.97)	0.162

Abbreviations: CAR: C-reactive protein-to-albumin ratio; FAR: fibrinogen-to-albumin ratio; NAR: neutrophil-to-albumin ratio; LAR: leukocyte-to-albumin ratio; NPAR: neutrophil percentage-to-albumin ratio; MAR: monocyte-to-albumin ratio; HALP: hemoglobin–albumin–lymphocyte–platelet score; m-HALP: modified HALP index; PNI: prognostic nutritional index; SII/Albumin: systemic immune-inflammation index-to-albumin ratio; SIRI/Albumin: systemic inflammatory response index-to-albumin ratio. *p* < 0.05 was considered statistically significant. * Adjusted for maternal age, pre-pregnancy body mass index, gravidity, parity, smoking status, gestational age at symptom onset, thyroid-stimulating hormone level, and prior history of hyperemesis gravidarum.

**Table 9:** Logistic regression analyses of inflammatory–nutritional and hematologic markers to predict re-hospitalization in hyperemesis gravidarum.

	Univariate OR (95% CI)	*p*-Value	Adjusted OR (95% CI) *	*p*-Value
CAR	1.78 (1.32–2.41)	<0.001	1.21 (0.86–1.70)	0.262
FAR	1.29 (1.01–1.66)	0.043	1.07 (0.82–1.39)	0.615
NAR	1.53 (1.22–1.91)	<0.001	1.19 (0.94–1.52)	0.147
LAR	0.88 (0.65–1.19)	0.402	—	—
NPAR	1.62 (1.28–2.04)	<0.001	1.33 (1.01–1.77)	0.043
MAR	1.31 (1.08–1.68)	0.017	1.09 (0.84–1.41)	0.512
SII/Alb	1.46 (1.19–1.89)	<0.001	1.18 (0.92–1.51)	0.180
SIRI/Alb	1.71 (1.30–2.21)	<0.001	1.26 (0.94–1.70)	0.115
HALP score	0.58 (0.46–0.73)	<0.001	0.71 (0.56–0.90)	0.005
m-HALP score	0.51 (0.39–0.66)	<0.001	0.66 (0.51–0.84)	0.001
PNI score	0.69 (0.58–0.82)	<0.001	0.83 (0.67–1.02)	0.058
Albumin	0.74 (0.61–0.86)	<0.001	0.88 (0.69–1.12)	0.301
Ketonuria level	1.84 (1.52–2.22)	<0.001	1.41 (1.10–1.81)	0.006
PUQE-24 score	1.39 (1.22–1.61)	<0.001	1.18 (1.01–1.39)	0.041

Abbreviations: CAR: C-reactive protein-to-albumin ratio; FAR: fibrinogen-to-albumin ratio; NAR: neutrophil-to-albumin ratio; LAR: leukocyte-to-albumin ratio; NPAR: neutrophil percentage-to-albumin ratio; MAR: monocyte-to-albumin ratio; HALP: hemoglobin–albumin–lymphocyte–platelet score; m-HALP: modified HALP index; PNI: prognostic nutritional index; SII/Alb: systemic immune-inflammation index-to-albumin ratio; SIRI/Alb: systemic inflammatory response index-to-albumin ratio; PUQE-24: Pregnancy-Unique Quantification of Emesis (24 h scale). *p* < 0.05 was considered statistically significant. * Adjusted for maternal age, pre-pregnancy body mass index, gravidity, parity, smoking status, gestational age at symptom onset, thyroid-stimulating hormone level, and prior history of hyperemesis gravidarum.

## Data Availability

The data supporting the findings of this study are available from the corresponding author (G.B.) upon reasonable request.
